# Beyond Received Wisdom and Authorised Accounts: What Knowledge Is Needed to Avoid Repeating History? Comment on "‘Attending to History’ in Major System Change in Healthcare in England: Specialist Cancer Surgery Service Reconfiguration"

**DOI:** 10.34172/ijhpm.2022.7647

**Published:** 2023-01-18

**Authors:** Graham P. Martin

**Affiliations:** University of Cambridge, Cambridge, UK.

**Keywords:** Change Management, Health Policy, Service Reconfiguration, Service Reorganisation, System Transformation

## Abstract

Perry and colleagues’ study of a programme to reconfigure cancer surgery provision in Greater Manchester highlights the importance of accounting for history in making successful change. In this short commentary, I expand on some of Perry and colleagues’ key findings. I note the way in which those leading change in Greater Manchester combined formal expertise in change management with sensitivity to local context, enhancing their approach to change through attention to details around relationships, events and assumptions that might otherwise have derailed the process. I identify lessons for others in how best to account for history in leading change, highlighting in particular the need to attempt to access and understand forms of history that may be suppressed, difficult-to-articulate, or otherwise marginalised.

 A recurrent finding of much research in health policy is how readily the lessons of the past are forgotten when planning or implementing change. ‘Zombie’ ideas—unproven or poorly evidenced policies and interventions that refuse to go away, and often return in slightly different clothing^1^—are a familiar feature of healthcare politics and policy. Some policy ideas are demonstrably ill-conceived and yet somehow remain irresistible.^2^ Others appear well founded in principle, but are blighted by repeated poor implementation.^3^ The difficulties that systems face in learning from the successes and failures of past change efforts have many roots—political obduracy, managerial churn, organisational amnesia—but addressing them effectively is a key challenge for improvement.

 Perry and colleagues’ account of the role of history in the apparently successful reconfiguration of specialised cancer surgery services in Great Manchester therefore offers a potentially valuable resource for those considering how best they can bring the lessons of the past to bear on their change efforts.^4^ Drawing on a wide range of contemporaneous qualitative data, Perry et al describe of the change process as it happened, from the perspectives of clinical and managerial stakeholders. Central to their argument is the contention that by “attending to history in terms of both personal and documentary accounts, those leading the reconfiguration of [oesophago-gastric] cancer surgery services in [Greater Manchester] developed a change process which took into account previous unsuccessful reconfiguration attempts, enabling them to reduce the impact of potentially challenging issues.”^4^ In particular, Perry et al draw attention to the importance of accounting for history in this reconfiguration work. History in their conceptualisation goes beyond the accepted record of what has happened and its impact on what is possible—‘history-as-fact,’ sometimes conveyed in the notion of path dependency—towards the need to understand the conflicts that lay behind what happened, and the prisms through which actors in the present understand what happened in the past—‘history-as-power’ and ‘history-as-sensemaking.’^4,5^ By accounting for the impacts of these histories, Perry et al argue, those leading change can shape the possibilities of the present more effectively. But this is not, they suggest, an Orwellian effort to seize control of the future by controlling the past^6^: rather, key to the apparent success of the work in Greater Manchester was a focus on creating the conditions for realising due process, rather than forcing through a preconceived outcome. In Perry and colleagues’ account at least, this was a matter of avoiding a repetition of the past by opening up possibilities, rather than an exercise in manipulation and imposition.

 The context facing those leading change in Perry and colleagues’ case study had some helpful features. Perhaps crucially, the history of past efforts at change here was comparatively uncontested: there were no fundamental disagreement on what had happened, even if there were different views about why. Moreover, there was widespread agreement that change of the kind envisaged was desirable, even necessary, based on a growing evidence base for the association between volume and outcomes in specialist surgery.^7^ This was not, then, a situation characterised by irreconcilable versions of the past, or incompatible visions for the future. A context receptive to change had also been fostered through the creation of the Greater Manchester Health and Social Care Partnership—part of a wider novel devolution of power to a regional authority that had itself been facilitated in part by a careful exercise in framing the history of the city region.^8^ This development, along with a broader trajectory in English health policy towards collaboration (and centralisation) over competition^9^—including extensive efforts to foster integration and shared decision-making processes in Greater Manchester specifically^10^—undoubtedly meant that those looking to reconfigure cancer surgery were working more fertile ground than their predecessors who had foundered.

 Nevertheless, much work remained if service reconfiguration was to fare better this time. Notable from Perry and colleagues’ account is the role played by an internal consultancy agency set up specifically for the purpose of leading on reconfiguration. One benefit of this approach was the way in which the inception of this body—the Transformation Unit—moved decision-making responsibility away, at least in appearance, from both commissioners and providers, and employed a carefully formulated stepwise approach to change. The work of the Transformation Unit drew on the usual repertoire of management consultancies supporting change^11^: deploying received forms of change management expertise; acting as a broker between organisations; running a carefully rehearsed but transparent and robust process that might forestall challenge; facilitating organisation development activity. Effective accounting for history on the part of those leading the programme, however, made a critical contribution to the apparent success of this strategy. Knowledge about the background, the relationships, the politics of cancer surgery in Greater Manchester allowed the Transformation Unit to apply its repertoire in an intelligent, context-sensitive manner—addressing potential challenges ranging from concerns as big as the historic distribution of power among provider organisations through to details as small as how to arrange chairs during engagement events to avoid personality clashes and maximise the chance of consensus.^4^ Effective application of the generic knowledge of change management was thus leavened by the insights offered by the specific knowledge of local history in a manner that mirrored, perhaps, the way the general knowledge base of evidence-based practice should be tailored, nuanced and enhanced by clinical judgement, shared decision-making and patient-centred care.^12^

 Particular forms and formats of history were particularly important in this work. While the facts of past reconfiguration efforts may have been relatively uncontested, attending to into the relational, emotional and political aspects of these attempts—in short, the subjective and inter-subjective—was critical. It was these details of history, so easily obscured in formal expositions of the past, that were crucial: these were the things that, if left unattended, had the potential to torpedo an effort that was otherwise exemplary in approach. Such details may matter disproportionately for the success of change. A reconfiguration that works in the abstract may fall if those on whose labour it rests do not wish to make it work in practice. Perhaps the key lesson from Perry and colleagues’ account for policymakers and practitioners in other contexts, then, is not to neglect these forms of history. A parallel might be drawn with the substantial body of research in organisational learning that describes the limitations of IT-based knowledge management systems. While these are often effective in storing and sharing formal and explicit knowledge, they are rarely able to capture, translate and transmit informal and tacit knowledge: know-how, shortcuts, relational knowledge.^13,14^ But such knowledge is fundamental to organisational effectiveness. Some forms of knowledge—rumour, gossip, speculation, allegation—are hidden, censored or even forbidden. Yet these too are an inescapable part of individual and organisational behaviour: discounting them will result in understanding that is partial at best.

 Just as effective organisations cannot rely on the efficient management of formal knowledge alone, so effectively accounting for history in change efforts must go beyond the formal and undisputed events of the past. Eliciting such knowledge is not easy.^15^ It is likely to involve extensive engagement with oral histories and personal viewpoints, some of them suppressed or devalued, rather than reliance on received documentary accounts alone. This may be important for both substantive and political reasons. Substantively, marginalised or undervalued perspectives may offer an understanding of what went wrong and what went right in change efforts of the past that are not visible from the ‘helicopter viewpoint’ of the powerful. Politically, while they may not have great formal power, these groups may be crucial in securing or denying implementation of change: if they do not buy in to what is being put forward, its success may be thwarted. The fuller their understanding of the multiple perspectives of relevant stakeholders on the past, the better equipped those leading change will be to open the possibilities of the future.

## Acknowledgement

 The author is based in The Healthcare Improvement Studies Institute (THIS Institute), University of Cambridge. THIS Institute is supported by the Health Foundation, an independent charity committed to bringing about better health and healthcare for people in the United Kingdom.

## Ethical issues

 Not applicable.

## Competing interests

 Author declares that he has no competing interests.

## Author’s contribution

 GPM is the single author of the paper.

## References

[1] Peters BG, Nagel ML. Zombie Ideas: Why Failed Policy Ideas Persist. Cambridge: Cambridge University Press; 2020. 10.1017/9781108921312.

[2] Alderwick H (2022). Health policy priorities for the next prime minister. BMJ.

[3] Powell M, Mannion R. “Groundhog Day”: the Coalition government’s quality and safety reforms. In: Exworthy M, Mannion R, Powell M, eds. Dismantling the NHS? Evaluating the Impact of Health Reforms. Bristol: Policy Press; 2016. p. 323-342.

[4] Perry C, Boaden RJ, Black GB, et al. “Attending to history” in major system change in healthcare in England: specialist cancer surgery service reconfiguration. Int J Health Policy Manag. 2022. 10.34172/ijhpm.2022.6389. PMC10105206 35297232

[5] Suddaby R, Foster WM (2017). History and organizational change. J Manage.

[6] Orwell G. Nineteen Eighty-Four. London: Secker & Warburg; 1949.

[7] Royal College of Surgeons. Position Statement: Reconfiguration of Surgical Services. London: RCS; 2018. https://www.rcseng.ac.uk/-/media/files/rcs/about-rcs/government-relations-consultation/rcs-position-paper-on-reconfiguration-september-2018-final.pdf.

[8] Haughton G, Deas I, Hincks S, Ward K (2016). Mythic Manchester: Devo Manc, the northern powerhouse and rebalancing the English economy. Camb J Reg Econ Soc.

[9] NHS England. Five Year Forward View. London: Department of Health; 2014. https://www.england.nhs.uk/wp-content/uploads/2014/10/5yfv-web.pdf.

[10] Walshe K, Lorne C, Coleman A, McDonald R, Turner A. Devolving Health and Social Care: Learning from Greater Manchester. Manchester: Alliance Manchester Business School; 2018. https://www.alliancembs.manchester.ac.uk/media/ambs/content-assets/documents/news/devolving-health-and-social-care-learning-from-greater-manchester.pdf.

[11] Kirkpatrick I, Sturdy AJ, Alvarado NR, Blanco-Oliver A, Veronesi G (2019). The impact of management consultants on public service efficiency. Policy Polit.

[12] Sackett DL, Rosenberg WM, Gray JA, Haynes RB, Richardson WS (1996). Evidence based medicine: what it is and what it isn’t. BMJ.

[13] Schultze U, Stabell C (2004). Knowing what you don’t know? Discourses and contradictions in knowledge management research. J Manag Stud.

[14] Swan J, Scarbrough H (2001). Knowledge management: concepts and controversies. J Manag Stud.

[15] Martin GP, McKee L, Dixon-Woods M (2015). Beyond metrics? Utilizing ‘soft intelligence’ for healthcare quality and safety. Soc Sci Med.

